# Professionally Delivered Local Antimicrobials in the Treatment of Patients with Periodontitis—A Narrative Review

**DOI:** 10.3390/dj9010002

**Published:** 2020-12-22

**Authors:** Amar Sholapurkar, Dileep Sharma, Beverley Glass, Catherine Miller, Alan Nimmo, Ernest Jennings

**Affiliations:** Clinical Dentistry and Oral Radiology, College of Medicine and Dentistry, James Cook University, Smithfield, Cairns, QLD 4878, Australia; dileep.sharma@jcu.edu.au (D.S.); beverley.glass@jcu.edu.au (B.G.); kate.miller1@jcu.edu.au (C.M.); alan.nimmo@jcu.edu.au (A.N.); ernest.jennings@jcu.edu.au (E.J.)

**Keywords:** local antimicrobials, local antibiotics, topical oral antibiotic, local drug delivery, periodontitis, periodontal therapy, periodontal treatment, scaling and root planning, scaling and root debridement

## Abstract

This review sheds light on the recent published scientific evidence relating to the use of professionally delivered local antimicrobial agents (LA’s). The review also analyses drug delivery systems available to date and provides an update on the latest scientific evidence about the benefits, limitations, and clinical results obtained by use of local drugs in the treatment of periodontal disease. The search strategy revealed randomized controlled trials (RCTs) that compared the efficacy of adjunctive LA’s to mechanical therapy alone. Based on the available evidence gathered from this review, we can infer that the use of local antimicrobial agents in conjunction to scaling and root debridement (SRD) delivers significant benefits in periodontal therapy and it is a useful aid, avoiding many of the side effects that systemic antibiotic therapy may involve. Local drug delivery (LDD) is an efficient and effective means of delivering drugs based on the evidence presented in the review. The authors of this review would suggest the use of local antimicrobials in cases of localized periodontitis or individual areas that do not respond to the usual mechanical therapy alone. This review summarizes the current use of local drug delivery in periodontal management ensuring that the general practitioners are able to choose an appropriate local antimicrobial.

## 1. Introduction

Periodontitis is a common disease of the oral cavity consisting of inflammation of the tooth supporting tissues, primarily caused by accumulation of complex polymicrobial dental plaque. It is initiated by Gram-negative tooth-associated microbial biofilms that elicit a host response, resulting in progressive, irreversible bone and soft tissue destruction (periodontal pocket formation, gingival recession or both), tooth mobility and exfoliation [1]. Genetic factors have been recently considered additional risk factor in the periodontal disease. It is also being investigated that gene-environment interactions are etiologically important in disease pathogenesis but the current knowledge in periodontitis is limited. The pro-inflammatory cytokine interleukin-1 (IL-1) is considered a key modulator of host responses to microbial infection and a major modulator of extracellular matrix catabolism and bone resorption, eventually leading to severe adult periodontitis [2]. Treatment to manage periodontal disease can involve the following: (1) mechanical debridement, which includes scaling and root debridement (SRD); (2) destruction of or interference with the metabolism of the organism, including the use of antibiotics and antiseptics; (3) affecting or altering the environment of the microorganisms associated with the periodontium/tooth interface [1]. Mechanical debridement, the most commonly used treatment modality in the management of periodontal disease has been quite successful in treating the majority of patients but carries a greater risk of recurrence when used alone, specifically in cases with systemic co-morbidities [1,3,4,5]. It has also been suggested that complete removal of plaque and calculus by mechanical debridement in inaccessible areas including deep pockets (more than 5 mm) and furcation areas is difficult, thereby leading to significant treatment failure rates [6].

Since SRD alone is insufficient to eliminate bacteria from the periodontal pocket, especially in inaccessible areas, antibiotics (both systemic and local) have been used as adjunctive agents in the management of periodontal disease for many years [3]. However, the repeated, long-term use of any systemic antibiotics may lead to potential complications, including risk of resistant strains, superimposed secondary infections, and possible lack of patient adherence [7]. Since periodontitis is a localized disease, local treatment is preferred over systemic therapy to avoid the complications associated with systematic administration of antibiotics. The key to success for periodontal therapy depends on the selection of an appropriate antimicrobial agent with appropriate route of drug administration. Minimal side effects to local drug delivery (LDD) and good patient adherence are other potential advantages compared to the systemic therapy [1,3,7]. Various studies have revealed that LDD into the periodontal pockets can provide higher therapeutic concentrations of the antibiotic compared to the systemic administration [1,8,9]. Local antibiotics including, tetracycline (TET), doxycycline (DOX), minocycline (MIN), metronidazole (MTZ), chlorhexidine (CHX), clarithromycin (CLM), azithromycin (AZM), moxifloxacin (MXF), clindamycin (CLI), and satranidazole (SZ) are presently being used in various drug delivery systems such as irrigations, fibres, films, injectable, gels, strips, compacts, vesicular liposomes, microparticles, and nanoparticle systems in the management of periodontal disease [4,7,8,9]. Therefore, the present review discusses the evidence from the past decade around the use of professionally applied local antimicrobial agents (LA’s) and highlights various delivery systems in the management of periodontal disease.

### 1.1. Various LDD Systems in the Treatment of Periodontal Disease

#### 1.1.1. Irrigation Devices/Systems

Oral irrigation (OI) can be described as a professionally employed irrigating system (used by dentists in the dental clinic) and those personally applied by the patients at home in order to prevent the periodontal disease [10]. Irrigation can easily eliminate the bacteria and its by-products from the periodontal pockets by constant water pressure on the tissue created by compressive force [10]. Several clinical studies have reported on the use of 0.6% triclosan, 1% polyhexamethylene guanidine phosphate, 10% povidone-iodine, 0.25% sodium hypochlorite, 0.75% boric acid, and 20 mg/mL concentration of ozonated water as irrigations [11,12,13,14,15,16,17,18,19]. The outcomes of the above studies were determined based on significant reduction in plaque index (PI), bleeding index (BI), periodontal pocket depth (PPD) scores, and clinical attachment level (CAL) gain. These irrigating agents, when used as an adjunct to SRP showed comparatively better therapeutic outcomes than in the control groups [11,12,13,14,15,16,17,18,19]. In general, it was found that the irrigating systems significantly showed good results but in the short-term since they have had a transient action. OI therefore had no significant long-term effect on that clinical parameters [10,11,12,13,14,15,16,17,18,19]. These limitations led to a search for more effective drug delivery systems such as fibres, films, strips, microspheres, and nanoparticles [10].

#### 1.1.2. Fibres

Fibres are reservoir-type systems, placed circumferentially into the periodontal pockets with an applicator and secured with cyanoacrylate adhesive or a periodontal dressing for the sustained release of drug into the pocket [13,20,21,22]. TET is a semi-synthetic, broad spectrum, bacteriostatic agent that interferes with bacterial protein synthesis and acts by inhibiting the tissue collagenase activity [23]. Several studies have been conducted in the past 10 years where investigators have incorporated TET drug in the fibres and have found positive clinical outcomes (gain in CAL and reduction in probing depths) in the treatment of periodontitis (Table 1). In 1994, the United States Food and Drug Administration (FDA) approved TET fibres for the treatment of adult periodontitis [10,20,21,22]. These are non-resorbable, biologically inert, safe, and composed of a plastic copolymer (ethylene and vinyl-acetate). The above system has now been unfortunately discontinued as a result of the polymer being non-biodegradable [13,20,21,22]. Periodontal Plus AB (TET impregnated collagen fibres) however has an added advantage of being bio-resorbable form of fibre for single application that usually biodegrades in the periodontal pocket within 7 days. Various studies have reported that local treatment with Periodontal Plus AB showed significant gain in CAL and reduction in probing depths compared to the control group [23,24,25,26]. The inter-comparison of clinical efficacy between TET-fibres and a xanthan based CHX gel (Chlosite^®^) showed that TET-fibres were better (they had significant gain in CAL and reduction in probing depths) than CHX gel [27].

#### 1.1.3. Matrix Delivery System—Films, Strips, and Chips

Films, strips, and chips are matrix delivery systems in which drugs are distributed uniformly throughout a polymer material, with controlled release occurring by either drug diffusion and/or matrix dissolution or erosion [20,21,22].

Periochip is the controlled subgingival delivery system which contains 2.5 mg of CHX gluconate (the concentration being 34% CHX), incorporated in a biodegradable matrix of hydrolysed cross linked gelatin with glutaraldehyde [10,20,21,22,28,29]. The advantage of this chip is that no second appointment is needed to remove it, as it is biodegradable. An in-vitro study has shown that PerioChip releases CHX in a biphasic manner, where it initially (in the first 24 h) releases approximately 40% of the drug followed by the release of the remainder of the drug in an almost linear fashion over 7–10 days [7]. PerioCol™-CG is another controlled-release CHX chip, which contains approximately 2.5 mg of CHX gluconate in a bio-degradable matrix of Type 1 collagen (a natural protein) derived from fish sources [20,21,22].

Several studies have been conducted in the past 10 years comparing the clinical and microbiologic efficacy by using adjunct locally delivered, controlled release antimicrobials in the form of films, strips, and chips (Table 2) [29,30,31,32,33,34,35]. The results of most of the studies revealed that adjunctive use of the Periochip or PerioCol™CG have resulted in a significant reduction of PPD and gain in CAL upon comparing the effect by SRD alone [29,30,31,32,33,34]. In another study, Lecic J et al. evaluated the clinical efficacy of different CHX gluconate preparations (CHX solution, CHX gel, and CHX chip respectively) applied subgingivally as an adjunct to SRD and found more favourable outcome in the clinical parameters when these preparations were used with SRD [35]. The most significant improvements were seen with the PI in the former 2 groups (CHX solution with SRD and CHX gel with SRD) at 1-month recall. In addition, there were improvements in the BI and PPD in the latter group (CHX chip with SRD) at 3-month recall.

#### 1.1.4. Gels

In periodontics, gels with active therapeutic agents (active pharmaceutical ingredient—API) are delivered into subgingival pocket gently with the use of wide port needle syringes which ensures an equal distribution of the drug [10]. Gels containing the antimicrobial agents are formulated using various polymers such as carbopol, xanthan, carboxy methyl cellulose, and chitosan. Various gel formulations have been developed e.g., Chlosite, DOX, MIN 0.5% CLM 0.5% AZM, MXF gel, MTZ gel, 3% SZ gel, and 1% CLI hydrochloride gel, by incorporating drugs at various concentrations [10,36,37,38,39,40,41,42,43,44,45,46,47,48,49,50,51,52,53].

Chlosite is a LDD containing 1.5% CHX of a xanthan (Ghimas Company, s.p.a, Bologna, Italy) gel matrix [10]. The gel dissolves within 10–30 days upon placement into the periodontal pocket which therefore helps maintaining the therapeutic concentration of the API for at least 15 days [10]. The gel matrix is mucoadhesive and therefore is not easily washed away by the flushing action of the gingival fluid or saliva. Table 3 provides detailed description of various gel forms, in the treatment of periodontitis. Various studies were designed to evaluate the clinical effects of topical application of xanthan-based Chlosite gel in randomized controlled clinical studies to treat periodontitis, to improve the effects of nonsurgical periodontal treatment in diabetic patients with periodontitis, and in a case series to treat periodontitis in smokers [36,37,38,39]. In all the above studies the subgingival injection of xanthan-based Chlosite^®^ gel adjunct with SRD appeared to cause significant improvement compared with SRD alone.

Doxycycline is a bacteriostatic antibiotic that demonstrates a wide spectrum of activity against common periodontal pathogens [40]. DOX levels in the periodontal pockets were found to be between 1500–2000 μg/mL in 2 h following local treatment. The levels of the drug remained above 1000 μg/mL at 18 h, after which it starting declining gradually [10]. Local application of DOX has resulted in several studies that reported the efficacy of DOX hyclate (10% DOX hyclate (Atridox^®^) as an antimicrobial agent for attaining PPD reduction and gaining the CAL [41,42,43].

Minocycline gel is usually available as 2% MIN hydrochloride in a matrix of 20 mg hydroxyethyl cellulose, 25 mg magnesium chloride, 10 mg eudragit, 60 mg triacetine, and 0.5 gm glycerine. A RCT evaluated the long-term efficacy of 2% MIN gel as an adjunct to SRD. The overall results however revealed no advantage over SRD and therefore the authors suggested further clinical trials for evaluation of its role as an adjuvant medication [47].

Clindamycin is a classic macrolide that has a broad spectrum of antimicrobial activity, better bioavailability, favourable tissue distribution, and a lower incidence of side effects [48]. It has been suggested that macrolides have the capability to penetrate the infected tissues at significantly higher concentration when compared to healthy tissues [48,49,50]. CLM is accumulated by phagocytes, monocytes, fibroblasts, polymorphonuclear cells, macrophages, and lymphocytes. Since these cells are more prevalent at periodontal disease sites, one could expect to see greater benefits [49]. Three different RCTs revealed that adjunctive use of subgingivally delivered 0.5% CLM as a controlled drug delivery system had a better clinical outcome compared to SRD alone [45,46,47].

Azithromycin is a semi-synthetic, acid stable antibiotic and represents the prototype of a novel class of macrolides called azalides. It has been shown to be efficacious against periodontal pathogens and also found to have significantly less bacterial resistance to the subgingival microflora [3,51,52]. However, the Azithromycin gel is not yet commercially available in the market for use. Three RCTs investigated effects of subgingivally delivered AZM gel; 0.5% concentration as an adjunct to SRD for treating chronic periodontitis [3,51,52]. Specifically, Agarwal E et al. focussed on patients with type 2 diabetes and AR Pradeep et al. investigated smokers [3,51]. In all the above trials SRD + 0.5% AZM gel showed enhanced reductions in PI, gingival index (GI), modified sulcus bleeding index (mSBI), and PPD and gains in CAL compared to the control group [3,51,52].

Moxifloxacin is a fourth generation fluoroquinolone antibiotic with a broad spectrum of antimicrobial activity. It exerts a bactericidal effect by specifically inhibiting adenosine triphosphate–dependent topoisomerase IV and topoisomerase II (DNA gyrase) [53]. Patients with periodontitis received a single subgingival application of different concentrations (0.125%, 0.4%, or 1.25%) of MXF in a gel or placebo gel immediately after full-mouth SRD. The 0.4% MXF gel group resulted in additional PD reduction compared to SRD alone [53].

Metronidazole is among the common antibiotics used in the treatment of periodontal disease [7]. Elyzol includes a MTZ 25% oil based, viscous, topical dental gel which has been used in the treatment of periodontitis over past few years [7]. Miani PK et al. concluded that 15% MTZ-based experimental gel group was superior to the control group in terms of lowering the bacterial counts after the intervention [54].

Satranidazole is another antibiotic that belongs to the 5-nitroimidazole group [55,56]. Two RCTs did report significant improvements (in subjects with periodontitis and type 2 diabetes) with adjunctive therapy (SRD + 3% SZ gel) compared to SRD alone [55,56].

#### 1.1.5. Micro Particulate System

Microparticles or microspheres (MC) are solid spherical polymeric structures having diameter between 1–1000 μm (it contains active drug) which are uniformly dispersed throughout a polymer matrix. This arrangement helps protect the drugs from external environment, masks any unpleasant taste, increases their bioavailability leading to constant drug action at the intended site [10,28]. A microparticle-based system consists of biodegradable polyalpha hydroxyl acids such as Poly Lactide (PLA) or Poly (Lactide-co-glycolite) PLGA in which the drug is encapsulated. This dissolves gradually, releasing an optimum concentration at the local site effectively [10]. Various drugs such as DOX, MIN, TET, and CLI have been used in the treatment of chronic periodontitis. Drugs like TET and MIN are usually delivered through microcapsules prepared from lactic acid/glycolic acid copolymers [28]. Adjunct locally delivered controlled release antimicrobials in the form of micro particulate systems have been consistently shown to be clinically effective and are described in Table 4.

Moura L.A. et al. investigated sustained release of LDD (DOX in PLGA MC) in the periodontal pockets and found promising results with significant decrease in gingival crevicular fluid (GCF) drug concentration (19.69 ± 4.70 μg/mL) that was assessed on the 20th day [57]. This meant that the concentration of the drug in the periodontal pocket stayed substantially high for 20 days which proved to be highly efficacious. Other investigations also showed that the LDD of DOX MC alone or by using Solid lipid microparticles (SLMs) encapsulating DOX hydrochloride and MTZ proved to be effective in the periodontal pocket therapy and reduced the *Porphyromonas gingivalis* counts significantly [58,59].

Minocycline: Arestin is the locally delivered, sustained release form of MIN MC (20–60 μm in diameter) as the hydrochloride [10]. Several studies concluded that MIN MC adjunct to SRD resulted in greater reduction of bleeding on probing (BOP) and PPD, higher CAL gain and decrease in the numbers and proportions of red complex bacteria [60,61,62]. On the other hand, Tabenski L. et al.’s 12-month prospective, RCT had completely contrasting outcomes [63]. They investigated whether any surplus effectivity was found in either antimicrobial photodynamic therapy (aPDT) or local application of MIN MC in the treatment of periodontitis following SRD. Surprisingly, neither of the systems showed any significant additional benefit in the management of periodontal disease. The authors believe that it might be due to a significant dropout rate during the study period and recruitment problems afterwards.

Tetracycline MC has also been incorporated as a drug in a micro particulate-based system. The mechanism of action of drug release is the same and it has been investigated that the release rate is influenced by the polymer choice (lactide/glycolide ratio), its molecular weight and crystallinity and the pH of the medium (TET release rate is increased as the pH increases) [10]. CLI-loaded microparticles on the other hand were investigated and appeared to have promising results for management of periodontal therapy [64].

#### 1.1.6. Nanoparticulate Drug Delivery System

Nanoparticles includes nanospheres and nanocapsules in the solid state, which are either amorphous or crystalline in nature and measuring approximately 10–200 nm in size [10]. They are meant to adsorb and/or encapsulate a drug, thus protecting it against chemical and enzymatic degradation [10]. In order to obtain an ideal delivery system for management of periodontal disease, triclosan-loaded polymeric (PLGA, poly-lactic-acid, and cellulose acetate phthalate) nanoparticles were prepared by an emulsification–diffusion process [10,28]. Madi M et al. evaluated and compared the anti-inflammatory effect of subgingivally delivered nanostructured DOX gel (nDOX) with conventional DOX gel used as adjunct to SRD. The former showed pronounced improvement in both clinical parameters and inflammatory markers at three months period [65].

#### 1.1.7. Liposome Systems

Liposome systems have been designed to mimic the bio-membranes in terms of structure and bio-behaviour and recently studied extensively in the treatment of periodontal diseases [66]. These are microscopic lipid based vesicles, which may either be unilamellar or multilamellar. These systems are prepared by using cholesterol, nontoxic surfactants, sphingolipids, glycolipids, long-chain fatty acids, and membrane proteins [64]. They have the property of being biocompatible, biodegradable, nontoxic, nonimmunogenic, highly stable and protect the drug from external environment [66,67]. However, on the other hand, they are also expensive to manufacture and have shorter half-life. It has also been found that these systems may lead to leakage and fusion of the encapsulated drug [68]. These devices work out much effective even in the deeper periodontal pockets in patients with severe periodontal disease [69]. In a recent study, Liu and co-workers conducted an experiment on rat periodontitis model and found that the liposome gel system containing 2% minocycline hydrochloride were much effective. The outcome of their study(by the end of 2, 4, and 8 weeks respectively) revealed reduction in the gingival index (GI), periodontal pocket depth, number of mononuclear cells, and odontoclasts. In addition to this, the investigators also observed new bone formation and fibres in the region of interest [70].

#### 1.1.8. Other Systems

AZM buccal patch—a recent investigation aimed to explore the clinical, microbiological, and biochemical impact of AZM buccal patch in chronic generalized patients, but the results concluded that AZM as monotherapy did not offer clinical benefits over SRD [71].

## 2. General Considerations of Various LDDs

The clinician needs to be well versed with the following factors that affect local drug delivery in periodontal pockets [72]. Firstly, the local antimicrobial agent should have the physical properties to reach the proposed site of action and be substantial, at a sufficient concentration for an extended period of time. The drug should therefore follow zero order kinetics to retain itself at the intended site for longer duration. In addition, the drug concentration at the site can be considerably affected by constant GCF flow/clearance. Secondly, the mode of drug delivery at the local area plays an important role in its efficacy. For example, the efficacy of the drug could be enhanced by the use of controlled drug release devices. However, subgingival irrigation of drug solutions in the periodontal pocket contributes to high concentrations for only short periods of time and therefore repeated OI is required to exhibit the proposed effect [72]. Moreover, since the biofilm formed in the periodontal pocket would usually interfere for the agent from diffusing into the soft tissue wall, it therefore needs to be disturbed first. Finally, an ideal drug should have a dose higher than minimal inhibitory concentration (MIC) in order to have its effect delivered at the local site [72].

Various antimicrobial agents are being used in irrigation systems and its success mainly depends on depth of penetration, virulence of bacteria, complexity of infection, the flow of GCF and concentration of the drug in the periodontal pocket over an extended period of time. In supragingival irrigation devices, the irrigating agent have the penetrating ability until the depth of 29–71% and 44–68% in shallow pockets and moderately to severe deep periodontal pockets respectively. On the other hand, the subgingival irrigation has better penetrability in the deep pockets (with the range of 75–93%). These irrigating agents when used as an adjunct to SRD showed comparatively better therapeutic outcomes than in the control groups but had no significant long-term effect on those clinical parameters [11,12,13,14,15,16,17,18,19].

Fibre-based systems used in the periodontal disease management appear to have some limitations in general [10]. Firstly; placement of fibres in the periodontal pocket requires more time (approximately 10 min) and considerable skills for the clinician. Moreover, these fibres may occasionally cause some discomfort to the patient, causing local erythema which could eventually interfere with the process of periodontal pocket healing [10].

Considering the use of matrix systems, following advantages have been reported [10,20,21,22]. The physical dimensions and shape of the films can easily be modified by measuring the dimensions of the pocket to be treated. There is minimal discomfort to the patient upon insertion of the film/chip in the periodontal pocket. Since the film/chip has good adhesive properties and the thickness does not exceed 400 µm, it will easily remain submerged into the periodontal pocket without any noticeable interference with the patient’s oral hygiene habits [10,20,21,22].

Gel formulations on the other hand have some advantages over other preparations. Gels can be more easily prepared and administered. They also have properties of higher biocompatibility and bioadhesivity allowing easy adhesion into the periodontal pocket, sustained drug release pattern, minimum dose frequency, and drug toxicity [10]. In all the studies (Table 3) the subgingival injection of xanthan-based Chlosite^®^ gel adjunct with SRD appeared to cause significant improvement compared with SRD alone [36,37,38,39]. However, the study by Calderini A. et al. indicated that CHX gel did not improve the treatment outcome over SRD alone [44]. Phogat M. et al. on the other hand found statistically significant comparable results upon use of xanthan-based CHX gel (Chlosite gel) and herbal extracts’ gel [45]. Furthermore, a study by Rusu D. et al. resulted in the products (CHX-based gingiva adhering gel containing herbal ingredients and 1% CHX water-soluble gel) having almost similar efficacies considering the clinical, microbiological and enzymatic outcomes at 3 and 6 months respectively after SRD [46]. DOX may be useful in diseases characterized by excessive collagen loss: it has the highest rate of reduction in the action of collagenase compared to MIN and TET [40]. Atridox is the only FDA approved gel system which is marketed as 8.5% DOX and available as 2 syringe mixing system [10]. Tomasi C et al. failed to get promising results for retreatment at molar furcation sites (which resulted in closure of only 50% of type I furcation sites and 17% of type II furcation sites respectively) with the use of 8.8% DOX gel [73].

The overall results associated with MIN gel revealed no significant advantage over SRD and therefore the authors suggested for further clinical trials for evaluation of its role as an adjuvant medication [44]. However, another study showed conflicting results (which demonstrated significant reduction in the clinical parameters with improvement in the periodontal status) using the same concentration of drug [74]. Furthermore, an investigation revealed that periodontal treatment with MIN gel improved the periodontal status and glycemic control with elevation of serum adiponectin in type 2 diabetic patients [75].

Upon local application of MTZ gel, the concentration of drug in the periodontal pockets was found to be above 100 μg/mL for at least 8 h which further declines to above 1 μg/mL concentrations at 36 h [7,10]. MTZ gel is also available as a bio absorbable delivery device consisting of MTZ benzoate distributed in a matrix containing glyceryl mono-oleate and sesame oil [76]. Upon contact with gingival crevicular fluid, MTZ gel forms reversed hexagonal liquid crystals which prevents the gel from easily spilling out from the periodontal pocket, thereby maintaining a MIC of the drug in the periodontal pockets for a long duration [76,77]. Bergamaschi et al. found no difference in the clinical and microbiological improvements when adjunctive MTZ (gel or tablet) was used in the management of periodontal disease [78].

The 2% MIN (in MIN MC) is encapsulated into bioresorbable polymer of polyglycolide-co-dl lactide which has resorption time of approximately 21 days [7,10]. The MC have an inert property of being bio adhesive upon contacting the moisture and therefore does not require additional adhesives or dressings to hold it in place. Once placed in the periodontal pocket, the MC react with the GCF crevicular fluid which hydrolyses the polymer and further releases MIN for approximately 14 days or longer before it resorbs completely [7,10].

The nanoparticulate system provides several advantages such as good dispersibility in an aqueous medium, controlled release rate and increased stability upon comparison with MC, microparticles, and emulsion-based delivery systems [7,10,28]. Due to their nano size, they tend to easily penetrate in regions of deep periodontal pockets that may not be accessible to other drug delivery systems. These systems also provide a uniform distribution of the active drug over long period of time [7,10,20,21,22,28]. Also, this system have the capability of being well absorbed and eventual bioavailability resulting in considerable reduction in drug dosage.

## 3. Conclusions

LDD is an efficient and effective means of delivering drugs based on the evidence presented in the review. The LDD in conjunction with SRD is effective in the treatment of localized periodontitis or in areas that do not respond to the usual mechanical therapy. LDD can achieve higher concentrations of the drug at the intended site of action by usage of lower dose, thus reducing the side effects. When considering the adjunctive use of these products clinicians should also consider other factors, such as the ease of handling, the time employed in its application, and its cost; all potentially influencing the overall efficiency of these therapies. At present, there is insufficient data to prove that one LDD system is superior to another. Therefore, the authors would also suggest researchers to conduct further large multicenter RCT with large sample size and longer follow-up of the patients to compare and determine the efficacy of these antimicrobials in the management of periodontitis. It is also worth mentioning to the clinicians to consider LDD’s as an adjuvant to conventional periodontal therapy to achieve best clinical results. 

## Figures and Tables

**Table 1:** Studies investigating role of various locally delivered fibre systems in the treatment of periodontal disease in the past 10 years.

Drug Delivery System Employed	Author, Year, and Ref. No	Drug Used	Trade Name of the Drug If Mentioned	Treatment Design	No. of Subjects in the Study	Duration of the Study (Days)	Major Outcome of the Study
Fibres	Meharwade et al., [24] 2014	TET impregnated collagen fibres	Periodontal Plus AB	Split mouth design study	90 sites from 30 patients	45	Local treatment with Periodontal Plus AB showed significant gain in CAL and reduction in probing depths. Also after treatment, GCF leptin levels significantly reduced in this group at day 15 but were increased almost to the pre-treatment levels on day 45 of evaluation. Concluded: nonsurgical periodontal therapies were not effective in maintaining stable reduction in the GCF leptin level even though there was significant reduction in pocket depths and CAL gain.
	F.Y. Khan et al. [24] (2015)	Resorbable collagen-based TET fibres	Periodontal Plus AB fibres	In-vivo study	40	90	Over the 3-month observational period, TET fibres demonstrated better results compared to the control group.
	Sachdeva S. et al. [23] (2011)	Biodegradable TET fibre	Periodontal Plus AB^TM^	Split mouth design	35	90	Combined antimicrobial and mechanical debridement therapy has shown better results as compared with SRD alone.
	Gill J.S. et al. [27] (2011)	TET fibres and a xanthan based CHX gel	Periodontal Plus AB® and Chlosite®	Randomized split mouth design	30	90	TET fibres are better (they had significant gain in CAL and reduction in probing depths) than CHX gel for treatment of chronic periodontitis.
	Shivojot Chhina et al. [26] (2015)	TET fibres	Periodontal Plus AB®	A RCT	30	90	More favourable outcome in both the clinical and biochemical variables when SRD was combined with LDD of TET fibres - reduction in the Alpha-2-macroglobulin levels in GCF.

**Table 2:** 10-year detailed summary of locally delivered controlled release antimicrobial matrix delivery systems.

Drug Delivery System Employed	Author, Year, and Ref. No.	Drug Used	Trade Name of the Drug If Mentioned	Treatment Design	No. of Subjects in the Study	Duration of the Study	Major Outcome of the Study
CHX chip	Konuganti K. et al. [29] (2016).	Flurbiprofen (FBP) and CHX chip	Not mentioned	RCT	50	180	Subgingival delivery of FBP or CHX chip as an adjuvant to SRD was more effective than SRD alone. Frequently applied CHX or FBP chips resulted in much more better outcome than single application.
	Gonzales J.R., et al. [30] (2011)	CHX chip—2.5 mg CHX gluconate	PerioChip, Dexcel Pharma.	RCT	24	180	Pre and post SRD application of CHX chips improved CAL and reduced the subgingival levels of the red complex microorganisms.
	Pattnaik S. et al. [31] (2015)	CHX gluconate chip	PerioCol™CG(Eucare Pharmaceuticals Pvt. Ltd., Chennai, India)	Clinico-microbiological study	20	90	PerioCol™CG showed better effects on the PPD, CAL and it eliminated most of the periodontal pathogens upon comparison with SRD alone.
	Kumar A.J., et al. [32] (2016)	CHX chip	(Periocol-CG, Eucare Pharmaceutical Pvt. Ltd. Thiruvakkam, Chennai, India)	RCT	30	90	Use of CHX chip had better outcome when compared with SRD alone.
	Grover V. et al. [33] (2011).	2.5 mg CHX gluconate chip	(Periocol CG; Eucare Pharmaceutical Pvt. Ltd., Chennai, India)	A clinical and radiographic study	40	90	PerioCol-CG was an effective adjunctive therapy to SRD.
	Lecic J. et al. [35] (2016)	CHX chip	Perio Chip^®^, (Perio Products, Jerusalem, Israel)	Randomized controlled, split mouth designed study	15	90	Use of CHX chip as an adjunct to SRD had better outcome when compared with SRD alone.

**Table 3:** Detailed study summary of investigated gel forms across the globe in the treatment of chronic periodontitis in the past 10 years.

Drug Delivery System Employed	Author, Year, and Ref. No.	Drug Used	Trade Name of the Drug If Mentioned	Treatment Design	No. of Subjects in the Study	Duration of the Study	Major Outcome of the Study
Xanthan-based Chlo-site gel	Jain M. et al. [36] (2013)	Xanthan-based Chlosite^®^ gel	Chlosite^®^ gel	RCT	30	180	Chlosite^®^ gel caused significant improvement compared with SRD alone.
	Matesanz P. et al. [37] (2013)	CHX formulations in a xanthan vehicle	(ChloSite^®^, Casalecchio di Reno, Bologna, Italy)	RCT	24	180	Adjunctive use of Xan–CHX improved the clinical outcomes to a limited extent, resulting with “residual” or “relapsing” pockets. No significant differences were detected between groups.
	Faramarzi M. et al. [38] (2017)	Xanthan- based 1.5% CHX gel.	CHLO-SITE^®^, (Ghimas, Italy)	RCT in diabetic patients	68	180	The investigators proposed that CHX gel might improve the effects of nonsurgical periodontal treatment in diabetic patients with periodontitis.
	Chandra C. and Chandra S. [39] (2010)	1.5% of CHX in 0.5ml of xanthan gel	Chlosite	A case series	74 sites from 3 chronic smoker patients	90	Combination of SRD and Chlosite resulted in additional clinical benefit upon comparison to the control groups.
	Calderini A. et al. [44] (2013)	Xanthan gel of CHX digluconate 0.5% and CHX dihydrochlor-ide gel 1%	Chlo Site; (Ghimas SpA, Bologna, Italy) Corsodyl gel; (GlaxoSmithKline SpA, Milano, Italy)	Preliminary case series	10	42	CHX gel formulation resulted in some additional benefits over SRD. On the other hand CHX gluconate had benefits but in the short term.
	Phogat M. et al. [45] (2014)	Xanthan- based CHX gel versus herbal extracts’ gel.	Chlosite gel, (GHIMAS, Italy)	RCT	150 sites from 30 patients	90	Investigation from Herbal gel were comparable to CHX gel in the treatment of chronic periodontitis.
	Rusu D. et al. [46] (2017).	(1) Hydrophobic gingiva adhering Gel with complex composition (2) Commercially available 1% CHX digluconate water soluble gel.	Chlorhexamed 1% gel; (Glaxo- SmithKline, Bretford, UK)	RCT	98	180	Both the groups had a relatively similar clinical, microbiological and enzymatic outcomes at 3 and 6 months after SRD respectively.

**Table 4:** Studies demonstrating use of microparticulate system in the management of periodontal disease in the past 10 years.

Drug Delivery System Employed	Author, Year, and Ref. No.	Drug Used	Trade Name of the Drug If Mentioned	Treatment Design	No. of Subjects in the Study	Duration of the Study	Major Outcome of the Study
DOX MC	Moura L.A. et al. [57] (2015)	Locally delivered DOX by poly (L-lactide-co-glycolide) (PLGA) MC	Not mentioned	Pilot study	19 periodontal pockets	20	On the 20^th^ day of evaluation, the researchers found a significant reduction in the concentration of drug in the GCF (19.69 ± 4.70 μg/mL). The DOX delivery system demonstrated a significantly good outcome in the patients with chronic periodontitis.
	Rao S.K. et al. [58] (2012)	DOX MC	Not mentioned	Parallel, single-blind, randomized, prospective study	14	180	DOX MC significantly led to improvement of clinical parameters and reduced P. gingivalis levels in the periodontal pocket.
	Gad H.A. et al. [59] (2017)	SLMs encapsulating DOX hydrochloride (DH) and MTZ	Not applicable since the gel was formulated in their pharmacy.	In-vitro and in-vivo clinical split mouth design study	12	14	Results revealed that the SLMs were safe to use and there was significant improvement in both microbiological and clinical parameters as compared to SRD alone.
MIN MC	Bland P.S. et al. [60] (2010)	MIN hydrochloride MC	Not mentioned	Multicenter, single-blind, randomized, parallel group, phase IV study.	127	30	Treatment using MIN MC greatly reduced the number of red complex bacteria and PPD significantly.
	Chiappe V.B. et al. [61] (2015)	MIN microgranules	Not mentioned	Randomized clinical and microbiological trial.	26	90	Results of this investigation revealed that MIN MC adjunct to SRD resulted in greater reduction of BOP and PD, higher CAL gain and decrease in the number of red complex bacteria, increased probability of Pg suppression and retarded recolonization of *Treponema denticola* when compared to SRD alone
	Srirangarajan S. et al. [62] (2011)	MIN MC and Commercially available MIN gel.	Atridox, Atrix Laboratories, Fort Collins, CO.	Randomized, split-mouth, single-masked study.	50	270	MC had a more sustained release and the initial drug concentration at the local site was high. There was also significant improvement in the PI and GI.
	Tabenski L. et al. [63] (2017)	aPDT and Local application of MIN MC	aPDT; Helbo^®^ Photodynamic Systems) and Local application of MIN MC (MC; Arestin, OraPharma)	RCT	45	365	Neither of the systems showed any significant additional benefit in the management of periodontal disease.
